# *COMT* Val^158^Met Polymorphism Is Associated with Verbal Working Memory in Neurofibromatosis Type 1

**DOI:** 10.3389/fnhum.2016.00334

**Published:** 2016-07-05

**Authors:** Danielle de Souza Costa, Jonas J. de Paula, Antonio M. Alvim-Soares, Patrícia A. Pereira, Leandro F. Malloy-Diniz, Luiz O. C. Rodrigues, Marco A. Romano-Silva, Débora M. de Miranda

**Affiliations:** ^1^Postgraduate Program in Molecular Medicine, School of Medicine, Federal University of Minas GeraisBelo Horizonte, Brazil; ^2^Department of Psychology, Faculty of Medical Sciences of Minas GeraisBelo Horizonte, Brazil; ^3^Department of Psychiatry, School of Medicine, Federal University of Minas GeraisBelo Horizonte, Brazil; ^4^National Institute of Science and Technology of Molecular MedicineBelo Horizonte, Brazil; ^5^Neurofibromatosis Outpatient Reference Center, School of Medicine, Federal University of Minas GeraisBelo Horizonte, Brazil; ^6^Department of Pediatrics, School of Medicine, Federal University of Minas GeraisBelo Horizonte, Brazil

**Keywords:** neurofibromatosis type I, *COMT* Val^158^Met polymorphism, working memory, arithmetic, genetic modifiers, neuropsychology, executive functions

## Abstract

Neurofibromatosis type I (NF1) is a neurogenetic disease marked by multiple cognitive and learning problems. Genetic variants may account for phenotypic variance in NF1. Here, we investigated the association between the catechol-*O*-methyltransferase (*COMT*) Val^158^Met polymorphism and working memory and arithmetic performance in 50 NF1 individuals. A significant association of the *COMT* polymorphism was observed only with verbal working memory, as measured by the backward digit-span task with an advantageous performance for Met/Met carriers. To study how genetic modifiers influence NF1 cognitive performance might be of importance to decrease the unpredictability of the cognitive profile among NF1 patients.

## Introduction

Neurofibromatosis type 1 (NF1) is a common neurogenetic disorder affecting 1 in each 3500 individuals (Friedman, 1999; Ferner, 2007). The *NF1* gene includes 63 exons and encodes a 220–250 kDa protein termed neurofibromin (Cawthon et al., 1990; Viskochil et al., 1990). NF1 is caused by mutations only in the *NF1* gene and have an autosomal dominant inheritance (Easton et al., 1993; Ward and Gutmann, 2005; Sabbagh et al., 2009). This single-gene disease is marked by cognitive, learning, and behavioral problems and is a potential model for the investigation of the biological mechanisms related to these complex phenotypes (Shilyansky et al., 2010).

Cognitive impairment and academic failure are the most common reported problems in the clinical care of NF1 individuals (Hyman et al., 2005). Executive function impairments impact overall academic achievement and quality of life with up to 80% of NF1 children experiencing moderate to severe deficits. NF1 affects planning, visuospatial processing, reading and vocabulary skills, and courses with an observed higher rate of attention-deficit/hyperactivity disorder and a mildly lower IQ score (Hachon et al., 2011; Lehtonen et al., 2013). However, there is a high variation among NF1 patients regarding the specific cognitive domain affected or the extension of the cognitive deficit (Lehtonen et al., 2013). In fact, NF1 phenotype varies from minimal to maximal presentation in all clinical characteristics, and cognitive and behavioral aspects are not an exception (Shilyansky et al., 2010).

Phenotype variability in NF1 is not easily explained. There are thousands of mutations described in the *NF1* gene with unsatisfactory genotype–phenotype associations (Pasmant et al., 2012). Even in the same family with multiple cases, a phenotypic variation of NF1 is present (Pasmant et al., 2012). It is possible that genetic variants also account for phenotypic variance in NF1 with the same mutation being modified concerning genotype–phenotype associations depending on different genetic backgrounds (Shilyansky et al., 2010).

Genetics has a significant influence on individual differences in cognitive function with dopamine-related polymorphisms among the most studied candidate genes (Savitz et al., 2006; Bellander et al., 2015). Dopamine level is essential for prefrontal function and cognition (Cools and Robbins, 2004), which is well documented for working memory and other aspects of cognitive control (Cools and D’Esposito, 2011). The catechol-*O*-methyltransferase (*COMT*) gene is the most investigated of the genes influencing dopamine-mediated functions (Dickinson and Elvevag, 2009). A commonly explored *COMT* variant, the Val^158^Met (rs4680), consists in a 158Val (*G*) to Met (*A*) polymorphism that reduces the activity of the COMT enzyme leading to a higher extracellular dopamine level mostly in the prefrontal cortex (PFC; Chen et al., 2004; Dickinson and Elvevag, 2009). Met allele carriers and conditions with intermediary values in a U-shape distribution of the dopaminergic synaptic availability in the PFC generally are favored in measures of cognitive control (Mier et al., 2010), though this is still a matter of controversy.

Different cognitive subprocesses may be differentially affected by the *COMT* alleles (Barnett et al., 2008; Mier et al., 2010). An example of the differential effect of the COMT alleles is on working memory. Working memory involves processes of maintenance and updating of information. It is an important cognitive function and is closely related to executive functions (Diamond, 2013). Regarding the *COMT* influence on working memory, performance requiring maintenance seems to be favored by the Met allele while the Val allele may be advantageous in updating tasks (Bellander et al., 2015). Testing different components of working memory (i.e., simple retention of information, content or modalities of information, and active manipulation of information) studies have shown that only mental manipulation of information is sensitive to the *COMT* dopaminergic modulation with Met/Met participants showing the best performance (Bruder et al., 2005; Aguilera et al., 2008). There are also investigations showing no significant association between the *COMT* gene and cognitive measures. Recently, a study using a multi-task approach found no effect of the COMT genotype on performance at highly demanding working memory loads (Ihne et al., 2016). Searching for evidence of a COMT genotype effect on working memory-related activation, a meta-analytic imaging study identified expected regions, namely the right inferior parietal lobe and the right dorsolateral PFC, as showing the highest likelihood for activation in both healthy controls and schizophrenia patients, but the significance of these results did not survive correction for a whole-brain approach (Nickl-Jockschat et al., 2015). On the other hand, many individual studies were able to find an association between the *COMT* alleles and performance in working memory tasks with activation of areas of the prefrontal–parietal–striatal network (Tan et al., 2007; Stokes et al., 2011; Kondo et al., 2015). Still, the association of the *COMT* gene with working memory is one of the best replicated so far (Mier et al., 2010; Ihne et al., 2016).

Cognitive impairment in NF1 has significant consequences in daily life, including prominent deficits in school abilities, which may occur in 75% of NF1 patients (Krab et al., 2008). Impairment in working memory and executive functions is a common feature of NF1, and might be an underlying contribute factor for the impairment in academic abilities (Hyman et al., 2005; Krab et al., 2008; Rowbotham et al., 2009). Working memory is highly involved in academic skills including reading, writing, and arithmetic (Baddeley, 2003; Geary, 2011). As stated before, working memory is a dopamine-mediated function. Dopamine homeostasis contributes to learning, memory, and attention, however, the mechanisms by which NF1 modulates dopamine signaling is still unknown (Diggs-Andrews and Gutmann, 2013). Therefore, in a multilevel perspective, *COMT* genotype (neurobiological level) might modulate working memory (cognitive level) and reflects on low academic achievement (functional level). To date, we found no study investigating the association of this specific genetic polymorphism with cognitive performance in an NF1 population. In this study, we aim to unravel the association between the *COMT* genotype, working memory performance, and school achievement (using a basic arithmetic test) in a heterogeneous NF1 sample. The study has the potential to provide insight into the mechanisms underlying phenotypic variability in NF1.

## Methods and Procedures

### Participants

Fifty participants with NF1 [19 subjects from 6- to 18-year-old (11.89 ± 4.11 years; 11 male) and 31 adults from 19- to 50-year-old (30.97 ± 8.81 years; 13 male)] were enrolled in this study. All individuals were recruited from a specialized clinic in neurofibromatosis at the Hospital of the Federal University of Minas Gerais. NF1 diagnosis followed the criteria specified by the National Institutes of Health statement [NIH] (1988) statement. Besides NF1, it was not reported by the participants or their families any history of genetic, neurological, or psychiatric disorders. This study is part of a research project that seeks to investigate molecular mechanisms of NF1 approved by the Federal University of Minas Gerais ethics committee. Written informed consent was obtained from all participants and/or from their parents according to the Declaration of Helsinki.

### Working Memory Assessment

All participants completed the age-appropriate digit-span subtest of the Wechsler Adult and Children Intelligence Scales (Wechsler, 2002, 2004) and the Corsi block-tapping task (Kessels et al., 2000, 2008). Both are span tasks where the examiner presents a growing sequence of numbers (digit-span) or moves on a wooden board (Corsi block-tapping). The subject must repeat the same sequence (*forward* versions of the tasks) or say/do it from the last to the first item (a *backward* version of the tasks). For each span (starting at two items), the examiner presented two different sequences. The tasks are stopped when the subject is not able to correctly repeat two sequences of same span length. We used the product of the maximum span length and number of correct trials as test measures (Kessels et al., 2008). This strategy usually produces more representative measures of working memory variability than the number of correct trials or the maximum span achieved.

### IQ Assessment

General intellectual functioning was assessed by the third version of the Brazilian Wechsler Intelligence Scales (WAIS-III or WISC-III for adults and children, respectively; Wechsler, 2002, 2004).

### School Performance Assessment

We adopted the arithmetic subtest from the School Achievement Test (Stein, 1994), as an objective measure of school performance. The School Achievement Test is a standard measure of academic skills including reading, writing, and arithmetic. The test was developed for the Brazilian population following the country educational agenda and have adequate normative data for grades 1–6. Participants’ scores on arithmetic were categorized in low-achievement or normal-high-achievement according to the guidelines proposed by Oliveira-Ferreira et al. (2012) and the total years of formal education showed by each participant.

### Socioeconomic Status Assessment

Socioeconomic status (SES) was assessed using the Brazilian Criterion for Economic Classification (CCEB) according to the criteria established by the Brazilian Research Enterprises Association (Associação Brasileira de Empresas de Pesquisa [ABEP], 2013). The CCEB estimates the purchasing power of families living in urban areas. It includes nine items that measure the available resources at home and one item that judges the education level of the householder, resulting in a scale ranging from 0 to 46 points, and segmentation into eight economic classes. These economic classes can be divided into three larger classes: “high” (A and B classes; median monthly household income from U$2349 to U$4152), “middle” (C class; median monthly household income from U$514 to U$1190) and “low” (D and E classes; median monthly household income of U$348). Eighteen NF1 participants (36%) were classified as high class, 27 (54%) as middle class, and five (10%) as low class.

### COMT Genotyping

The polymorphism was assessed by a standard procedure previously reported (Pereira et al., 2012). Genomic DNA was extracted from blood samples using the high salt method (Lahiri and Nurnberger, 1991). The COMT functional polymorphism (val158met, *rs4680*) was purchased in a made-to-order from Applied Biosystems^®^. Genotyping was performed using a real-time PCR system in the allelic discrimination mode (Stratagene Mx3005 – MxPro QPCR-Software, 2007) using the TaqMan Genotyping Master Mix (Applied Biosystems, Foster City, CA, USA). PCR parameters included an initial denaturation at 95°C for 10 min, followed by 50 cycles at 95°C for 15 s and 60°C for 1 min. Each reaction contained 3.5 μl of mix, 0.1 μl of the probe, 3.4 μl of deionized water, and 1.0 μl of DNA. Researchers involved in genotyping were blind to neuropsychological results, and researchers participating in neuropsychological assessments were blind to the genotyping results. COMT genotype was coded as a categorical variable (Val/Val, Met/Val, and Met/Met) for further analysis.

### Statistical Procedures

Most of our data was non-normally distributed. The use of data transformation procedures (square, cube, square root, and logarithm) did not succeed in normalizing the data distribution. We then adopted non-parametric tests for the following procedures. Non-parametric univariate comparisons performed by the Kruskal–Wallis tests did not show differences between age (χ^2^ = 3.21, *p* = 0.201), years of formal education (χ^2^ = 0.65, *p* = 0.721), SES (χ^2^ = 1.03, *p* = 0.596), or intelligence (χ^2^ = 1.83, *p* = 0.400) between the genotype groups. In this sense, we compared the three *COMT* genotypes (Val/Val, Val/Met, Met/Met) in the digit-span and Corsi block-tapping tasks by the same statistical procedure. To ensure results’ consistency, we analyzed the *p*-values along with the effect sizes (“*r*” conversion computed by dividing the resulting “*Z*” by the square root of the total sample size). This method can be interpreted as a correlational coefficient, and effect sizes higher than 0.3 can be considered moderate and larger than 0.5 interpreted as large according to Cohen’s (1988) guidelines. *Post hoc* comparisons between each *COMT* genotype were corrected by the Dunn–Bonferroni method. The comparison between the two groups defined by the school achievement and the *COMT* genotype was performed by a chi-square test. A secondary analysis investigated the association between *COMT* genotype, IQ, and working memory with arithmetic’s performance. We stratified the participants based on the School Achievement Test performance and used multinomial stepwise logistic regression models to assess whether low school achievement was associated with neurobiological and cognitive measures. All statistical procedures were performed in SPSS 20.0.

## Results

**FIGURE 1:**
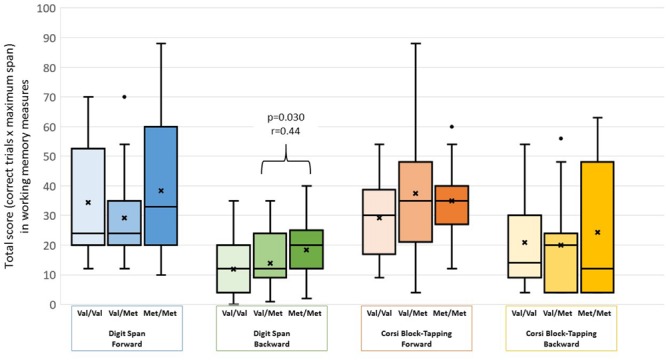
**Box-plots showing participants performance on working memory measures stratified by COMT genotype.** We found no significant differences in the digit-span forward (χ^2^ = 1.06, *p* = 0.587), Corsi block-tapping task forward (χ^2^ = 4.29, *p* = 0.117) and backward (χ^2^ = 3.27, *p* = 0.195). In the digit-span backward condition, significant group differences were found (χ^2^ = 6.65, *p* = 0.036). The Met/Met group outperformed the Val/Met group (*Z* = 2.58, *p* = 0.030, *r* = 0.44) but not the Val/Val group (*Z* = 0.87, *p* = 1.000). There were no differences between the Val/Val and Val/Met groups (*Z* = 1.69, *p* = 0.273). “x” represents test means. The dots represent outlier observation points.

Demographic and cognitive characteristics of the participants are shown in **Table 1**. There were no differences between the genotype groups regarding sociodemographic features. No significant differences in performance according to *COMT* genotype were found for the digit-span forward (χ^2^ = 1.06, *p* = 0.587), Corsi block-tapping task forward (χ^2^ = 4.29, *p* = 0.117) and backward (χ^2^ = 3.27, *p* = 0.195). In the digit-span backward condition, significant group differences were found (χ^2^ = 6.65, *p* = 0.036). The Met/Met group outperformed the Val/Met group (*Z* = 2.58, *p* = 0.030, *r* = 0.44) but not the Val/Val group (*Z* = 0.87, *p* = 0.999). There was no difference in performance between the Val/Val and Val/Met groups (*Z* = 1.69, *p* = 0.273). Group differences are represented in **Figure 1**.

**Table 1:** Demographic and cognitive characteristics of the participants.

	Val/Val (*N* = 16)			Val/Met (*N* = 23)			Met/Met (*N* = 11)			KW^1^	
	Pc.25	Pc.50	Pc.75	Pc.25	Pc.50	Pc.75	Pc.25	Pc.50	Pc.75	χ^2^	*p*
Age (years)	17	26	32	10	19	29	18	28	44	3.21	0.201
Formal education (years)	8	9	11	3	11	11	8	9	11	0.65	0.721
Socioeconomic status	16	21	26	15	19	25	16	20	33	1.03	0.596
Full scale IQ	89	95	103	77	94	106	89	98	106	1.73	0.400
Digit-span (forward)	22	31	47	18	24	35	20	33	35	1.06	0.587
Digit-span (backward)^∗^	9	12	20	4	9	20	12	20	25	6.65	0.036
Corsi block-tapping (forward)	34	35	44	16	30	40	35	40	48	4.29	0.117
Corsi block-tapping (backward)	14	21	38	4	12	24	4	18	48	3.27	0.195

*^1^Kruskal–Wallis Test, Pc=Percentile, ^∗^significant at *p* < 0.05.*

The school performance analysis showed that 40% of our sample had difficulties in basic arithmetic skills according to the cut-offs of the School Achievement Test (i.e., performance below the 25 percentile). However, we found no significant difference between low-achievement and normal-high-achievement groups regarding *COMT* genotypes distribution (χ^2^ = 0.952, *p* = 0.621). The final step of the backward logistic regression model was significant (χ^2^ = 26.30, df = 2, *p* < 0.001) and showed a moderate sensitivity (83%) and specificity (75%) for individual classification. IQ (*p* = 0.007) and working memory assessed by the backward digit-span task (*p* = 0.020) were directly associated with lower arithmetic performance, but not *COMT* genotype neither the remaining working memory measures.

## Discussion

Our preliminary results support an advantageous working memory performance in NF1 Met/Met carriers, which strengthens the hypothesis of genetic variants accounting for phenotypic variability in NF1. Considering the well-established COMT polymorphism effect on working memory (Mier et al., 2010; Bellander et al., 2015), we add into this line of evidence showing a *COMT* Val^158^Met genotype effect on cognitive control even in a sample of subjects with a monogenic disorder with compromising of behavior and cognition.

The COMT effect on working memory in our sample, however, was only observed for performance on the backward condition of the digit-span task. This result is in line with studies showing a COMT effect on measures demanding an active process of manipulation, but not on measures that only require maintenance of information (Bruder et al., 2005; Aguilera et al., 2008). Therefore, the lack of a COMT influence on the forward conditions of the working memory tasks that we observed is not without precedents. The absence of a COMT association with the backward version of the Corsi block-tapping task could lead us to hypothesize about a content-dependent (i.e., verbal vs. visuospatial) effect. Nevertheless, other studies have not shown such modality-dependent differences (Bruder et al., 2005; Aguilera et al., 2008; Ihne et al., 2016). Moreover, the backward Corsi block-tapping task have failed to demonstrate the same level of difficulty compared to the backward digit-span task and participants reach the same performance on both the forward and the backward versions of the Corsi block-tapping task (Mammarella and Cornoldi, 2005; Kessels et al., 2008). Thus, it seems more likely that our findings reflect a major COMT influence on measures demanding greater mental manipulation of information in working memory (Bruder et al., 2005).

In two different animal models of NF1, an inverted relation between the reduction of dopamine levels and the impairments of spatial learning were observed (Anastasaki et al., 2015) suggesting the importance of dopamine activity for NF1 cognition. It has been shown that neurofibromin modulates inhibitory networks in prefrontal and striatal regions, impacting working memory performance (Shilyansky et al., 2010), but our results suggest that variability in cognitive level expression between NF1 individuals may occur as a result of variability in their genetic background. It has been hypothesized that genetic modifiers could interact on a more functional level to exacerbate or compensate for the signaling changes caused by loss of *NF1* (Shilyansky et al., 2010). Future studies are needed to show whether NF1 may moderate known effects of other specific genetic polymorphisms on cognition.

Although we have found no direct effect of the COMT genotype on NF1 arithmetic performance, the backward digit-span was predictive of lower arithmetic performance, together with IQ, in our sample. It is important to emphasize that 40% of the subjects in this study were classified as showing difficulties in basic arithmetic abilities. Working memory is known to be important for numerical processing (González-Giraldo et al., 2014). Despite controversies regarding which allele would be advantageous to numerical abilities, the *COMT* Val^158^Met has been associated with arithmetical functioning (Júlio-Costa et al., 2013; González-Giraldo et al., 2014) with dopamine playing a key role in updating new information at the neural systems level (Tan et al., 2007). It is possible that the lack of association at a functional level (academic performance) with the COMT polymorphism in our study reflects a bias of sample power, but future studies are needed to investigate the existence of a more direct effect of *COMT* on arithmetic in NF1.

To our knowledge, this is the first study finding associations of a polymorphism in the *COMT* gene with cognitive measures in NF1 participants. This result may have practical implications since it may add evidence to the usefulness of dopamine-targeted therapies for some NF1 individuals with executive impairments (Diggs-Andrews and Gutmann, 2013). For example, the pharmacological response of NF1 individuals to methylphenidate, a psychostimulant medication that increases extracellular dopamine availability in dopaminergic neurons, might results in improved attention and working memory and consequent better academic performance (Mautner et al., 2002; Lion-François et al., 2014). In this sense, the genetic background may be useful to determine the sensibility of specific groups to distinct therapy methods. Additionally, to study how genetic modifiers influence NF1 cognitive performance might be of importance to decrease the unpredictability of the cognitive profile among NF1 patients. In this context, we have to emphasize that it is a preliminary data in a small sample, but with some potential consequences. In conclusion, we found a preliminary data identifying modifier genes such as COMT polymorphism being associated with working memory performance in an NF1 sample.

## Author Contributions

Conceived and designed the experiments: DS, JP, DM, and MR-S. Neuropsychological data collection and supervision: DS, JP, and LM-D. Clinical NF1 data collection and supervision: DM and LR. Genetic data analysis: AA-S and PP. Analyzed the data: DS and JP. Contributed reagents/materials/analysis tools: DM, LM-D, MR-S, and LR. Wrote the paper: DS, JP, AA-S, PP, LR, LM-D, MR-S, and DM.

## Conflict of Interest Statement

The authors declare that the research was conducted in the absence of any commercial or financial relationships that could be construed as a potential conflict of interest.
